# White sponge nevus of the oral cavity: Affecting members of two generations in a family

**DOI:** 10.1002/ccr3.7082

**Published:** 2023-03-08

**Authors:** Abhishek Gupta, Ram Sudan Lamichhane, Anju Redhu

**Affiliations:** ^1^ Department of Oral Medicine and Radiology Chitwan Medical College Bharatpur Nepal; ^2^ Department of Oral Pathology KIST Medical College and Teaching Hospital Lalitpur Nepal; ^3^ Department of Oral Medicine and Radiology PGIDS Rohtak India

**Keywords:** autosomal dominant, Cannon's disease, oral mucosa, white lesion, white sponge nevus

## Abstract

The typical feature is the autosomal dominant heritance and clinically dormant, non‐scarpalble, white diffuse, soft, thickened white plaques with a corrugated surface affecting mostly the buccal mucosa bilaterally which can substantially lead to the diagnosis of white sponge nevus.

## Graphical abstract

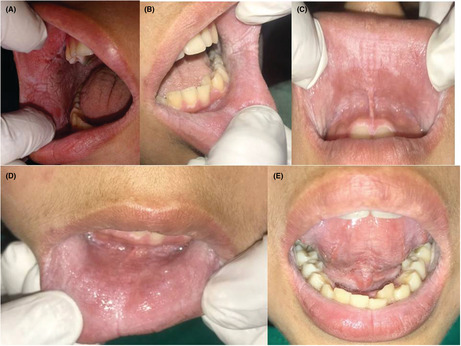

## 1 INTRODUCTION

White sponge nevus (WSN) is a rare, autosomal dominant hereditary disorder with a high degree of penetrance and variable expression.^1^ It is characterized by asymptomatic spongy white plaques that affect any part of the oral mucosa and less frequently the nasal, esophageal, rectal, and genital mucosa.^1^ Oral WSN appears as white or gray diffuse plaques thickened with multiple furrows and spongy texture.^1^, ^2^ The diagnosis of WSN can be made with proper history and careful clinical examination and histopathological examination can be done only to confirm the diagnosis.^3^ After its first mentioned in 1909 by Hyde, the detailed reporting was done by Canon in 1935 who coined the term WVN.^4^ Since then many cases have been reported and a prevalence of below 1 in a 200,000 have been documented in literature making it a rare entity.^4^, ^5^ No cases have been reported to date in Nepal.

## 2 CASE HISTORY/EXAMINATION

**FIGURE 1:**
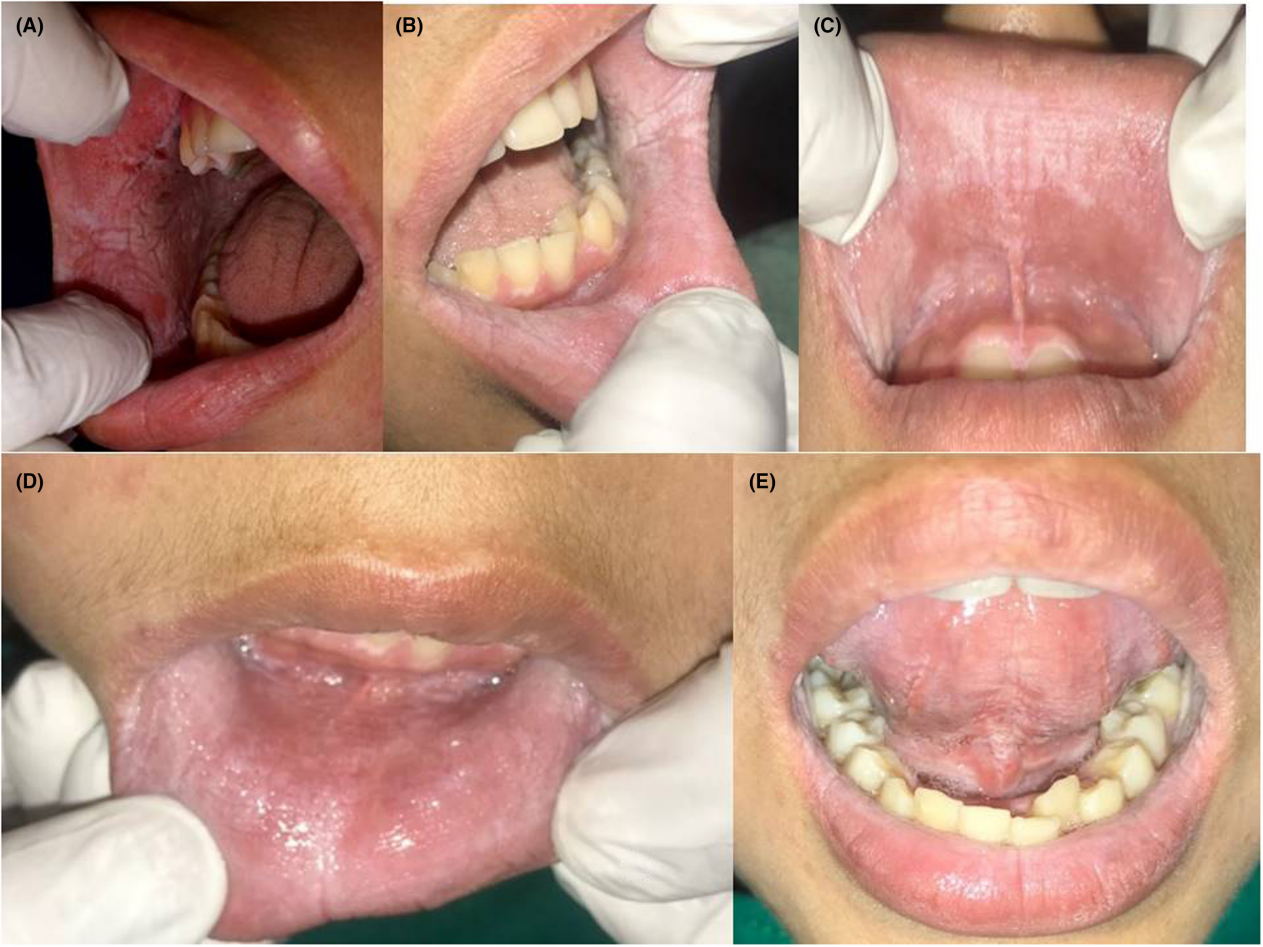
White spongy plaques irregular in shape noted with respect to bilateral the buccal mucosa (A, B); upper and lower labial mucosa (C, D); ventral surface of the tongue and floor of the mouth (E).

A 14‐year‐old female patient presented to the Department of Oral Medicine and Radiology with chief complain of sensitivity on intake of cold water with respect to right lower back teeth region since 20 days. Intraoral examination revealed occlusal caries with respect to mandibular right first molar and was nontender, on percussion. A striking incidental finding was noted on performing her intraoral examination. There were asymptomatic, bilateral, white spongy plaques irregular in shape noted in the buccal mucosa, upper and lower labial mucosa, ventral surface of the tongue, and floor of the mouth (Figure 1A–E).

**FIGURE 2:**
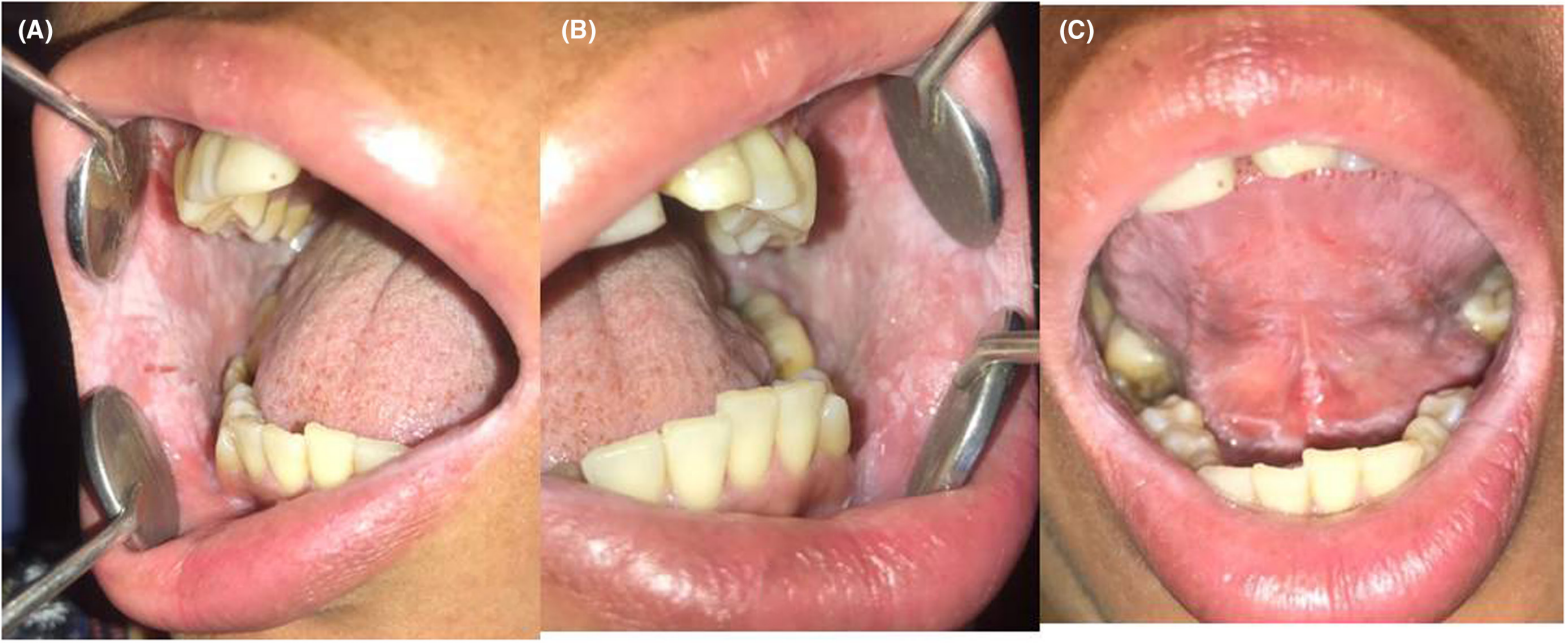
White spongy plaques irregular in shape noted in the buccal mucosa (A, B); ventral surface of the tongue and floor of the mouth (C).

There was no erythema associated with the plaques, but a generalized peculiar opalescent hue was present. On palpation, it was soft in consistency, nontender, and did not wipe off. The surface appeared smooth, thickened, folded, and corrugated with velvety texture. No lymph nodes were palpable. There was no relevant past medical history with no history of consanguinity. On further evaluation, the patient's mother reported similar lesions in her oral cavity. On examination, similar lesions were noted with respect to bilateral buccal mucosa and ventral surface of the tongue in the 36‐year‐old mother (Figure 2A–C).

The patient and her mother both were asymptomatic but reported a history of irregular use of topical antifungal agents (prescribed by dentists) since the initial presence of the lesion with no improvement or reduction in the size of the lesion to date. They were not worried about the lesion as according to them they have been living with it since childhood, also the mother reported similar lesions in her mother. Patients were not willing for a biopsy, so it could not be carried out. Moreover keeping in mind the insignificance contribution of biopsy to the treatment plan, the cost, and trauma involved, the diagnosis of WSN was given, which was purely on the basis of history and clinical findings of the duo. The patients were referred to the Department of Ophthalmology and Obstetrics/Gynecology to rule out the presence of conjunctival and genital lesions. Therapeutic management was not needed as the lesions were asymptomatic.

## 3 DISCUSSION

White sponge nevus is considered a rare genetic disorder, affecting 1 in 200,000 people.^3^ Our report has described the first two cases from Nepal. It is associated with mutations in the genes that encode for mucosal specific keratins K13 and K4 expressed in the spinous layer of the oral epithelium.^4^ In the present case, the disease was transmitted from the affected mother to her daughter, consistent with an autosomal dominant mode of inheritance. Some authors have reported no gender predilection and some have reported of female predominance (3:1 ratio).^2^, ^3^ In the present case, the two members of two generations affected were only females.

The characteristic clinical presentation of WSN is that of white plaques in the oral cavity, most predominantly affecting the buccal mucosa.^5^ But in the present case less common intraoral sites like the tongue, labial mucosa, and floor of the mouth were involved in the daughter.

Prominent hyperkeratosis, marked acanthosis, spongiosis, cleared cytoplasm with perinuclear eosinophilic condensation are a common histological characteristic feature.^6^ Histological findings are characteristics but not pathognomonic to the lesion. Also, our patient did not agree for biopsy so it was not performed. The clinical appearance of WSN may have overlapping features with a variety of oral lesions that have varied clinical behaviors and treatment protocols, hence it is important to distinguish.

The differential diagnosis includes a diversity of processes (benign, malignant, chemical) (Table 1).

**TABLE 1:** Differential diagnosis of WSN.

	Lesion	Distinguishing features
Benign	Focal (Frictional) Keratosis	A white, keratotic lesion due to chronic mechanical irritation caused by sharp edges of teeth or restorations, oral appliances (denture, orthodontic), abrasive foods, vigorous tooth brushing, and playing wind instruments
	Dyskeratosis congenita	Usually associated with hematological abnormalities along with white patches of oral cavity, reticulate skin hyperpigmentation and nail dystrophy.^6^, ^7^ Predispostion to malignancy.^6^, ^7^
	Pachyonychia congenita	Characterized mainly by hypertrophy of the nails and hyperkeratosis of the skin and mucosae.^8^
	Acute pseudomembranous candidiasis (Oral thrush)	Patchy white plaques or flecks on mucosal surface, scrapping usually reveal area of erythema or shallow ulceration
	Leukoedema	Gray‐white, diffuse, filmy, or milky surface, bilateral buccal mucosa. A developmental phenomenon that is more common in darker‐skin individuals and Disappears with stretching the mucosa.
	Hereditary benign intraepithelial dyskeratosis	Early onset (usually within the first year of life) of bulbar conjunctivitis and oral white lesions, Soft, asymptomatic, white folds and plaques of spongy oral mucosa, usually sparing the tongue.
	Darier disease (Follicular keratitis)	Hyperkeratosis palmaris et plantaris, Fingernail changes may include fragility, splintering, and subungual keratosis. Favored oral mucosal sites include the attached gingiva and hard palate. The lesions typically appear as small, whitish papules, producing an overall cobblestone appearance.
	Focal epithelial hyperplasia	Occurrence of multiple or unique whitish or normal in color small papules or nodules in oral cavity, especially on labial and buccal mucosa, lower lip and tongue, and less often on the upper lip, gingiva and palate^9^
	Oral hairy leukoplakia	A white plaque with corrugated surface, painless, and not removable by scraping Commonly found in the lateral border of the tongue.
Oral potentially malignant disorders	Oral Lichen Planus‐ Plaque type	White straie and other types of lichen planus found in association.
	Leukoplakia	A predominantly white, non‐wipable lesion of the oral mucosa having excluded clinically, histopathologically or by the use of other diagnostic aids other, well‐defined predominantly white lesions
Malignant	Oral florid papillomatosis (Verrucous carcinoma)	Multiple verruciform and papillomatous growths that converge forming plaques and vegetations Considered as a variant of low degree of malignancy of oral mucosal verrucous carcinoma^10^
Chemical	Chemical burns	Caused by topical applications of medications/therapeutics (aspirin, adalimumab, dental products, etc) and various foods (notably garlic). A grayish‐white fibrin‐coated ulcer at the site of application associated with burning sensation/pain.

Based on the history, clinical presentation, and after ruling out other white lesions, we arrived at the diagnosis of WSN.

To date, only 1 case of malignant transformation has been reported which was attributed to the chronic use of prednisone.^8^ No success has been achieved with (a) Conservative management of beta‐carotene, antibiotics, antihistamines, retinoic acid, tetracycline mouth rinse and (b) surgical intervention by Resection or laser ablation.^9^ However, the patient has been kept under regular follow‐up.

## 4 CONCLUSION

In conclusion, first the attending clinician should try to establish etiopathologic correlations with the list of processes in differential diagnosis and then consider performing a biopsy to confirm the diagnosis and also to rule out possible malignancy, particularly for patients with history of use of tobacco product and/or alcohol. If a patient is asymptomatic and does not desire any treatment or is unconcerned about the lesion, we strongly recommend to keep the patient on regular follow‐up for any changes. This case report is presented for its rarity and also its resemblance to other white lesions. Hence, it is the duty of an oral physician to correctly diagnose the condition to avoid unnecessary treatment.

## AUTHOR CONTRIBUTIONS

**Abhishek Gupta:** Conceptualization; formal analysis; methodology; project administration; resources; supervision; validation; visualization; writing – original draft; writing – review and editing. **Ram Sudhan Lamichhane:** Conceptualization; formal analysis; supervision; validation; writing – review and editing. **ANJU REDHU:** Conceptualization; formal analysis; supervision; writing – original draft; writing – review and editing.

## FUNDING INFORMATION

There were no sources of funding.

## CONFLICT OF INTEREST STATEMENT

The authors declare that there is no conflict of interests regarding the publication of this paper.

## CONSENT

Written Consent was taken from the patient and patient's guardian.

## Data Availability

The data are available with the correspondence author and can be availed on request.
